# Comparison Between Mohindra Retinoscopy and Wet Retinoscopy in Refraction of Infants and Children

**DOI:** 10.18502/jovr.v21.17651

**Published:** 2026-09-17

**Authors:** Mousffa Abdul Ghafoor, Afia Tahir, Shahid Noor, Zaneera Hassan, Iffa Raees, Ruhullah

**Affiliations:** ^1^College of Ophthalmology and Allied Vision Sciences, King Edward Medical University, Lahore, Pakistan; ^2^Department of Ophthalmology, Fazle Omar Hospital, Rabwah, Pakistan; ^3^Department of Optometry, The University of Faisalabad, Faisalabad, Pakistan; ^4^Department of Optometry and Vision Sciences, The University of Lahore, Lahore, Pakistan

**Keywords:** Children, Infants, Mohindra Retinoscopy, Refractive Error, Wet Retinoscopy

## Abstract

**Purpose:**

The purpose of this study was to compare the results of Mohindra near retinoscopy and wet retinoscopy in the refraction of infants and children.

**Methods:**

This comparative cross-sectional analytical study included 56 participants, comprising two equal groups of 28 infants and 28 children with refractive errors. Both Mohindra near retinoscopy and wet retinoscopy were performed on each infant and child. Mohindra near retinoscopy was performed first in a dark room at a distance of 50 cm with one eye occluded. Wet retinoscopy was performed after Mohindra near retinoscopy using 1% cyclopentolate hydrochloride eye drops. The Wilcoxon signed-rank test was used for comparison between the two techniques.

**Results:**

The mean spherical values obtained by Mohindra near retinoscopy were 0.14 ± 2.97 D and 0.20 ± 3.04 D for the right and left eyes, respectively, whereas wet retinoscopy demonstrated higher spherical means of 0.52 ± 3.28 D and 0.58 ± 3.17 D. Statistically significant differences were observed between Mohindra near retinoscopy and wet retinoscopy for spherical measurements in the right (*Z* = –3.665, *P* < 0.001) and left (*Z* = –3.600, *P* < 0.001) eyes. Significant differences were also found in cylindrical measurements for the right (*Z* = –2.380, *P* = 0.017) and left (*Z* = –3.355, *P* = 0.001) eyes.

**Conclusion:**

Wet retinoscopy yielded statistically significant differences in refractive measurements compared to Mohindra near retinoscopy in infants and children. Mohindra near retinoscopy underestimated hyperopic and astigmatic refractive errors. These findings suggest that wet retinoscopy remains the fundamental method for pediatric refractive assessment, particularly in infants and in cases of latent hyperopia.

##  INTRODUCTION

Refractive errors are the main cause of visual impairment among school-age children and one of the most common causes of treatable blindness.^[1]^ Uncorrected refractive errors are the leading cause of visual impairment (43%), followed by cataracts (33%).^[2,3]^ Numerous studies have investigated the global prevalence of refractive errors.^[4,5]^ In Pakistan, studies have reported the prevalence of refractive errors among school-age children to range from 8.9% to 19.8%.^[2,6]^ Cycloplegic refraction, also known as wet retinoscopy, is the gold standard for pediatric refractive assessment. Cycloplegia is essential in children because the high amplitude of accommodation may mask latent hyperopia and other accommodative errors.^[7]^

Wet retinoscopy is particularly useful in young, uncooperative, or non-communicative patients and in cases with suspected accommodative abnormalities, latent hyperopia, and esotropia.^[8,9]^ Cycloplegia is commonly achieved using agents such as cyclopentolate, tropicamide, and atropine, which inhibit accommodation by blocking muscarinic receptors in the ciliary body.^[10,11]^ Mohindra near retinoscopy is a non-cycloplegic technique used for refracting young children without the use of cycloplegic drops. The examination is performed in a dark room at a working distance of 50 cm with monocular fixation to reduce accommodative stimulus.^[12]^ The accommodative response during Mohindra near retinoscopy has been reported to stabilize around 0.70 D in adults.^[13]^ This technique may be useful in children who are anxious about cycloplegic drops, require frequent follow-up examinations, or have a history of adverse reactions to cycloplegic agents.^[14]^ Mohindra near retinoscopy differs from dynamic retinoscopy because cycloplegia is not applied during the examination.^[15]^ Previous studies comparing Mohindra near retinoscopy and cycloplegic refraction primarily focused on children older than 1 year of age and mainly evaluated spherical refractive measurements. Therefore, a significant gap is still present regarding the comparison of these techniques in infants and children.

This study aimed to compare the results of Mohindra near retinoscopy and wet retinoscopy in refraction of infants and children, and to evaluate the differences between the two techniques in pediatric refractive assessment.

##  METHODS

### Study Design and Setting

This comparative cross-sectional analytical study was conducted at the Eye Department of Fazle Omar Hospital, Pakistan. A total of 56 participants were enrolled, comprising two equal groups of 28 infants and 28 children with refractive errors. The study adhered to the tenets of the Declaration of Helsinki and the applicable ethical guidelines in Pakistan, and it was approved by the Ethics Committee of Fazle Omar Hospital, Pakistan (Code of Ethics No. 602).

### Participant Selection

Participants were divided into two age groups, including 28 participants in each group: infants ( < 1 year of age) and children (1-8 years of age). A non-probability sampling method was used. The study population consisted of 30 boys and 26 girls. The inclusion criteria comprised infants and children aged 3 months to 8 years, irrespective of gender. Children with strabismus were also included. Infants and children with intellectual disability and other ocular conditions were excluded.

### Data Collection Procedure

Mohindra near retinoscopy was performed first in a dark room at a distance of 50 cm. One eye was occluded while the other eye was tested. Refractive errors were neutralized with the help of trial lenses. The final prescription was adjusted by subtracting the empirically derived value of 1.75 D. Following Mohindra retinoscopy, wet retinoscopy was done by using drops of 1% cyclopentolate hydrochloride.

One drop was instilled into each eye, followed by a second drop 10 minutes later. Wet retinoscopy was performed 45-50 minutes after instillation of the first drop in a semi-dark room at a working distance of 1/3 meter. The final prescription was adjusted by subtracting the working distance of 1.50 D and a tonus allowance of 0.50 D. Each participant underwent refractive assessment using both techniques. Mohindra near retinoscopy was performed under monocular conditions, whereas wet retinoscopy was performed following cycloplegia.

#### Instruments

• Pen torch
• Retinoscope
• Near fixation target
• Distance fixation target
• Trial box

### Statistical Analysis

All data were analyzed using the Statistical Package for Social Sciences (SPSS version 23.00). Quantitative variables were expressed as mean ± standard deviation (SD) for descriptive purposes. Normality of the variables was assessed using the Shapiro-Wilk test. As most variables were not normally distributed (P < 0.05), non-parametric statistical methods were applied. The Wilcoxon signed-rank test was used to compare refractive measurements obtained by Mohindra near retinoscopy and wet retinoscopy. Statistical significance was set at P < 0.05.

**Table 1:** Demographic characteristics of the study participants (*n* = 56)

**Variable**	**Category**	***n*** ** (%)**
Age group	Infants ≤ 1 years	28 (50.0)
	0-4 months	7 (12.5)
	4-8 months	11 (19.6)
	8-12 months	10 (17.9)
	Children ≤ 8 years	28 (50.0)
	1-4 years	15 (26.8)
	4-8 years	13 (23.2)
Gender	Boys	30 (53.6)
	Girls	26 (46.4)
Total		56 (100.0)
*n*, number.		

**Figure 1:**
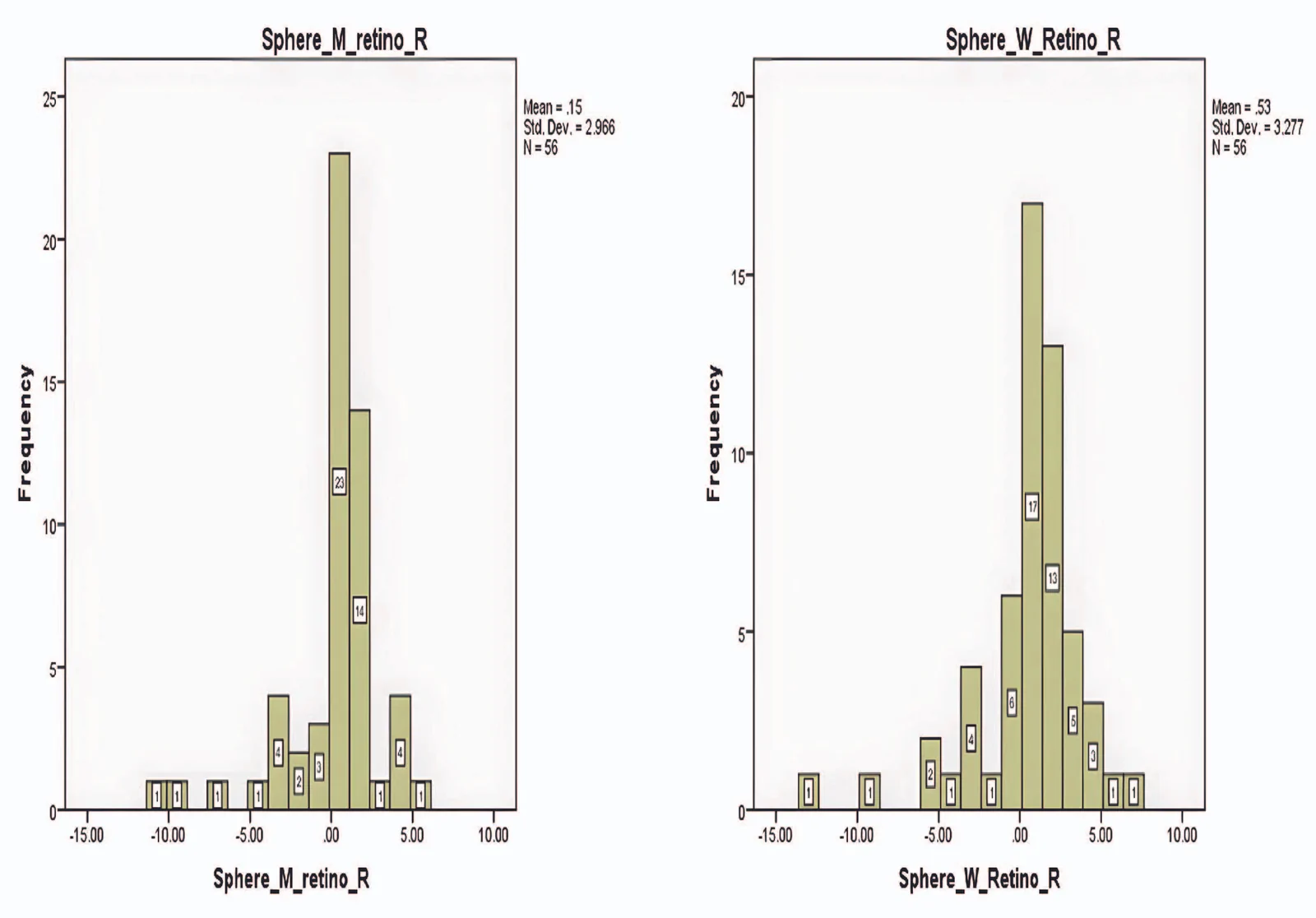
The two graphs compare the right eye's spherical values measured by Mohindra retinoscopy and wet retinoscopy, respectively. The *X*-axis shows spherical values, and the *Y*-axis shows frequency.

**Figure 2:**
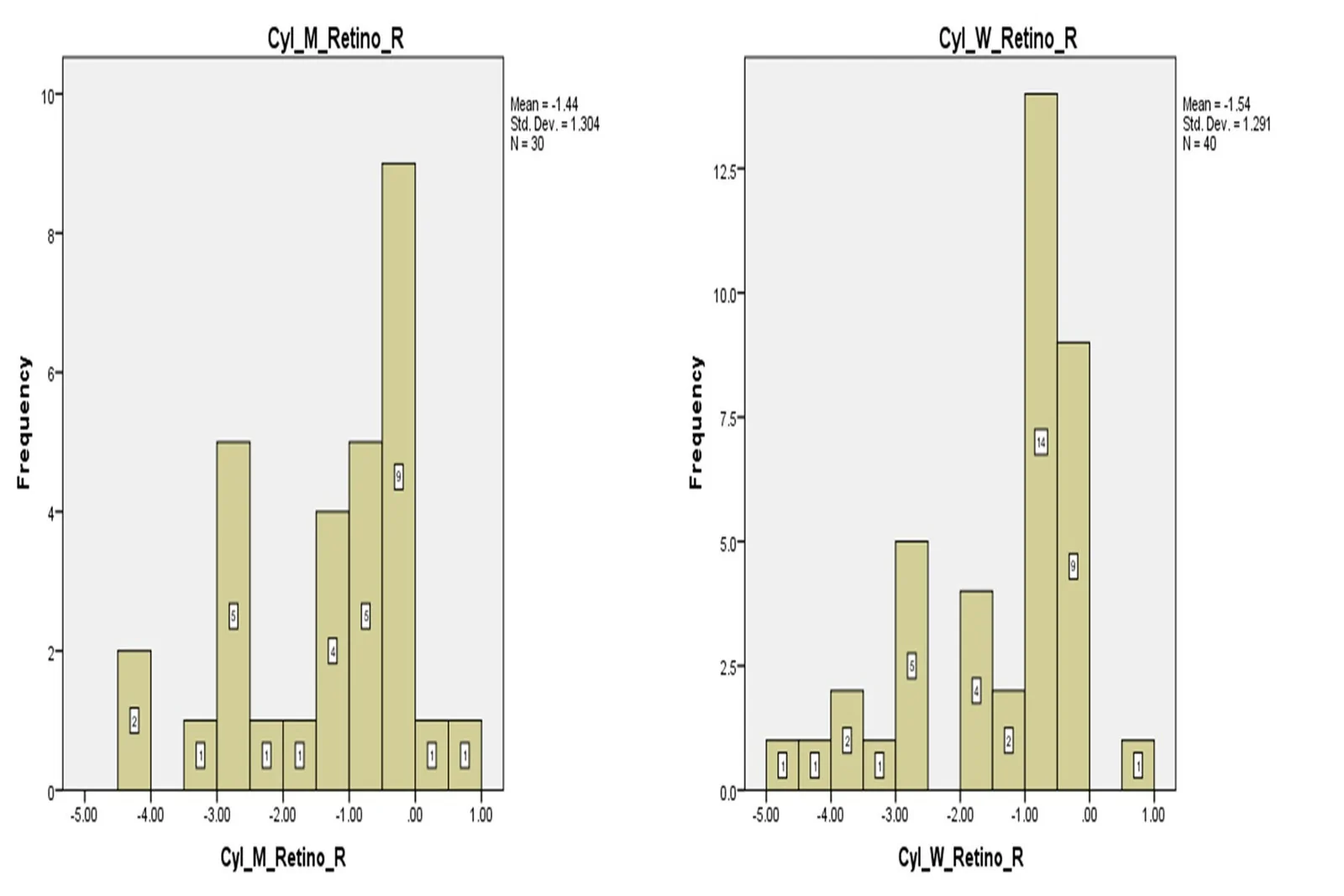
The two graphs compare the right eye's cylindrical values measured by Mohindra retinoscopy and wet retinoscopy, respectively. The *X*-axis shows cylindrical values, and the *Y*-axis shows frequency.

**Figure 3:**
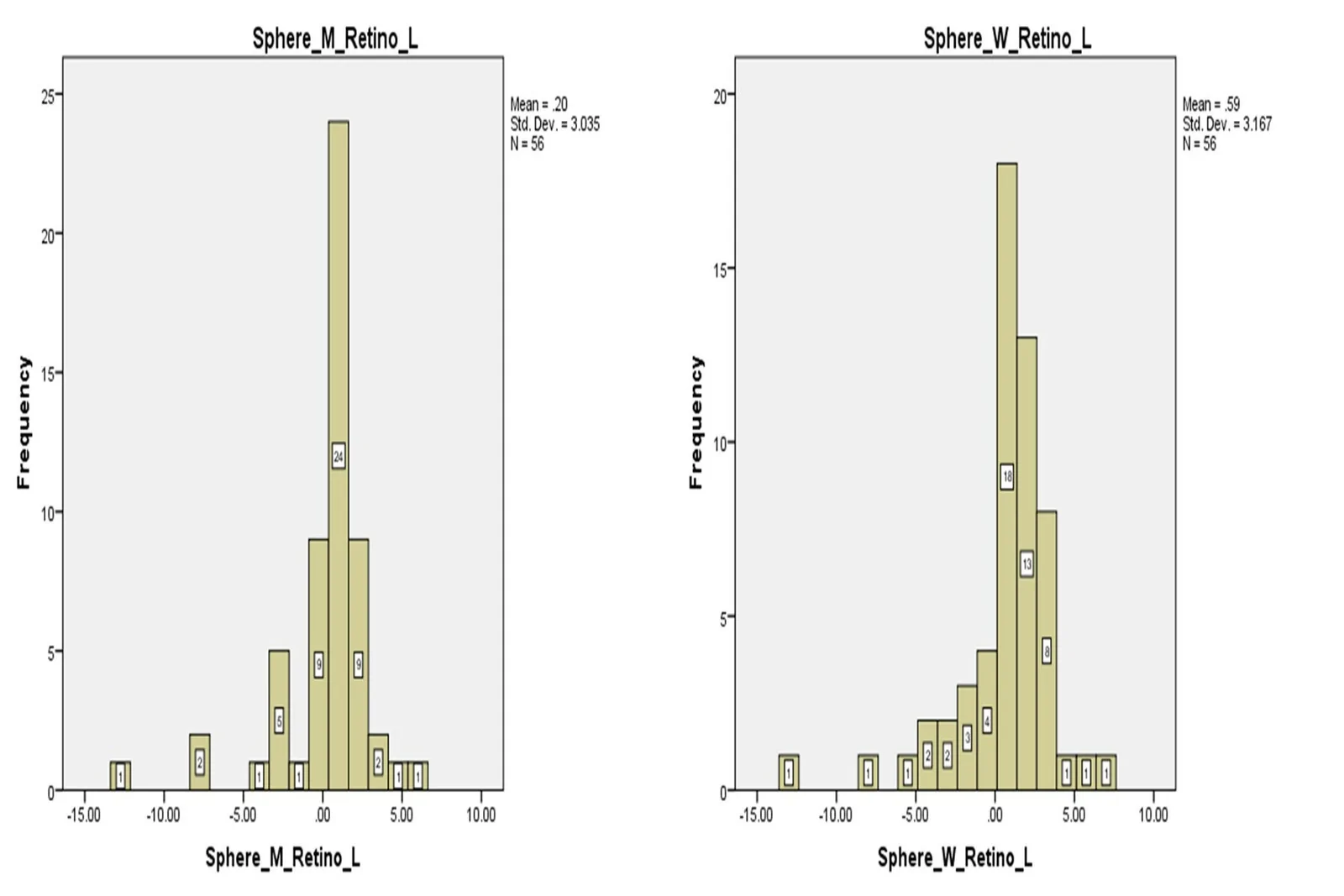
The two graphs compare the left eye's spherical values measured by Mohindra retinoscopy and wet retinoscopy, respectively. The *X*-axis shows spherical values, and the *Y*-axis shows frequency.

**Figure 4:**
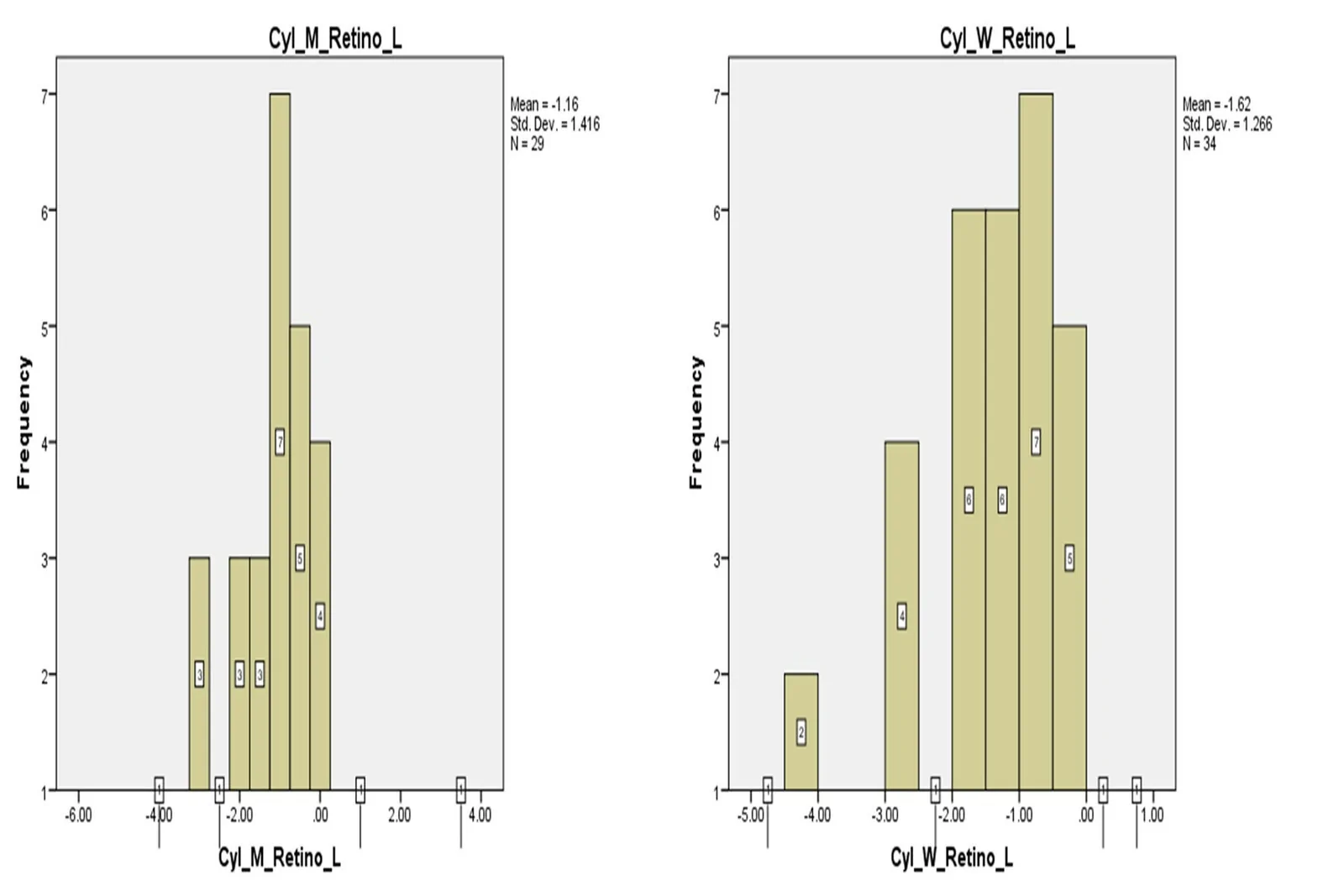
The two graphs compare the left eye's cylindrical values measured by Mohindra retinoscopy and wet retinoscopy, respectively. The *X*-axis shows cylindrical values, and the *Y*-axis shows frequency.

**Table 2:** Descriptive statistics of refractive measurements obtained by Mohindra near retinoscopy and wet retinoscopy

**Method**	**Parameter**	**Eye**	***N***	**Mean ± SD**	**Range**
	Spherical	R	56	0.1473 ± 2.97 D	–10.75 to +5.00 D
	Spherical	L	56	0.2009 ± 3.04 D	–12.75 to +5.75 D
MNR	Cylindrical	R	30	–1.4417 ± 1.30 D	–4.25 to +0.50 D
	Cylindrical	L	29	–1.1638 ± 1.42 D	–4.00 to +3.50 D
	Spherical	R	56	0.5268 ± 3.28 D	–13.00 to +6.50 D
	Spherical	L	56	0.5893 ± 3.17 D	–13.00 to +6.50 D
WR	Cylindrical	R	40	–1.5438 ± 1.29 D	–4.75 to +0.75 D
	Cylindrical	L	34	–1.6176 ± 1.27 D	–4.75 to +0.75 D
MNR, Mohindra near retinoscopy; WR, wet retinoscopy; R, right eye; L, left eye.					

**Table 3:** Comparison of wet retinoscopy and Mohindra near retinoscopy using the Wilcoxon signed-rank test

**Parameter**	**Mean difference (WR – MNR)**	***Z*** **-value**	***P*** **-value**
Spherical power (RE)	0.38 D	–3.665	0.001
Cylindrical power (RE)	–0.10 D	–2.380	0.017
Spherical power (LE)	0.39 D	–3.600	0.001
Cylindrical power (LE)	–0.45 D	–3.355	0.001
MNR, Mohindra near retinoscopy; WR, wet retinoscopy; RE, right eye; LE, left eye. The mean difference was calculated as the difference between the mean values obtained by wet retinoscopy and Mohindra near retinoscopy (WR – MNR).			

##  RESULTS 

A total of 56 participants ranging from 3 months to 8 years old (mean age, 3.3 ± 1.4 years) were evaluated in two equal groups of 28 infants and 28 children. Of the total number of participants, 28 (50.0%) were under 1 year of age, and 28 (50.0%) were up to 8 years of age. Of these, 30 (53.6%) were boys and 26 (46.4%) were girls. The participant demographics are outlined in Table 1.

The spherical value of the right eye refracted by Mohindra near retinoscopy ranged from –3.00 D to +3.00 D. On the other hand, the spherical value of the right eyes found by cycloplegic (wet) retinoscopy ranged from +0.00 D to +3.00 D in most participants [Figure 1].

The results of Mohindra near retinoscopy showed that 30 (53.6%) out of 56 participants had cylindrical refractive error in the right eye, and most values ranged from –0.25 D to –1.50 D. The axis of the right eyes found by Mohindra near retinoscopy showed that the majority of participants, 23 (76.6%), had with-the-rule astigmatism. The results of wet retinoscopy showed that 40 (71.4%) out of 56 participants had astigmatism in the right eye, mostly ranging from –0.50 D to –1.00 D [Figure 2]. The axis of the right eyes obtained by wet retinoscopy showed that the majority of participants, 33 (82.5%), had with-the-rule astigmatism.

Mohindra near retinoscopy showed that most participants had a spherical value between +2.75 D and –3.00 D in the left eye. Using wet retinoscopy, we found that this value ranged between –2.25 D and +3.00 D in the left eye in most participants [Figure 3].

Mohindra near retinoscopy showed that 29 (51.8%) out of 56 participants had cylindrical numbers in their left eyes. Most participants had cylindrical values between –0.25 D and –2.00 D. The axis of the left eyes found by Mohindra near retinoscopy showed that the majority of participants, 25 (86.2%), had with-the-rule astigmatism. Wet retinoscopy showed that 34 (60.7%) out of 56 participants had astigmatism in their left eye, with most values ranging between –0.50 D and –3.00 D [Figure 4]. The axis of the left eyes found by wet retinoscopy showed that the majority of participants, 30 (88.2%), had with-the-rule astigmatism.

Descriptive statistics for the retinoscopy measurements are summarized in Table 2. Wet retinoscopy demonstrated higher mean spherical and cylindrical measurements compared with Mohindra near retinoscopy in both eyes.

Comparison of wet retinoscopy and Mohindra near retinoscopy is displayed in Table 3. The test revealed a statistically significant difference between the spherical values obtained by Mohindra near retinoscopy and wet retinoscopy (*Z* = –3.665, *P* < 0.001) for the right eyes, and (*Z* = –3.600, *P* < 0.001) for the left eyes. Furthermore, a statistically significant difference was observed between the cylindrical values obtained by Mohindra near retinoscopy and wet retinoscopy (*Z* = –2.380, *P* = 0.017) for the right eyes, and (*Z* = –3.355, *P* = 0.001) for the left eyes.

##  DISCUSSION 

A total of 56 participants, comprising two equal groups of 28 infants and 28 children with refractive errors, were examined in this study. Both retinoscopy techniques were performed on each infant and child. The findings demonstrated statistically significant differences between the two techniques for both spherical and cylindrical measurements. Wet retinoscopy generally yielded higher hyperopic spherical measurements and greater cylindrical measurements than Mohindra near retinoscopy. The observed differences may be attributed to the effect of cycloplegia on accommodation. Children possess a high amplitude of accommodation that may influence refractive findings during non-cycloplegic examination. By minimizing accommodative activity, wet retinoscopy may reveal refractive errors that could otherwise remain partially masked during Mohindra near retinoscopy. This may explain the relatively higher hyperopic and astigmatic measurements obtained with wet retinoscopy in this study. The statistically significant *Z*-values and *P*-values indicate that Mohindra near retinoscopy consistently underestimates spherical refractive errors in both eyes when compared to wet retinoscopy. Specifically, Mohindra near retinoscopy tends to yield more myopic or less hyperopic spherical values. Similarly, Mohindra near retinoscopy also underestimates cylindrical powers, thereby detecting less astigmatism than wet retinoscopy. The findings of the current study are generally consistent with previous studies that have evaluated the agreement and correlation between Mohindra near retinoscopy and cycloplegic refraction in children and reported that both techniques may provide comparable findings under certain conditions. However, less agreement has been reported in cases of latent hyperopia.^[16,17]^ In contrast, the present study included infants under 1 year of age and comprehensively evaluated both spherical and cylindrical refractive measurements.

Infants have a large amplitude of accommodation and may continue to accommodate even when one eye is occluded, leading to variability in refractive measurements. This accommodative instability may contribute to an underestimation of hyperopia during Mohindra near retinoscopy. In addition, maintaining stable fixation in this age group is often challenging. Some infants may be uncooperative during examination, making it difficult to obtain consistent refractive measurements. Such factors may reduce the precision of Mohindra near retinoscopy. In contrast, wet retinoscopy is performed following the administration of cycloplegic eye drops, which helps minimize accommodative influences during examination. Although the instillation of cycloplegic drops may cause temporary agitation and crying in some infants and children, most patients are relatively cooperative during retinoscopy once adequate cycloplegia has been achieved. If cycloplegia is incomplete, infants may continue to accommodate during retinoscopy, resulting in variable refractive measurements. Therefore, wet retinoscopy should be performed only after adequate cycloplegia has been achieved to ensure optimal refractive assessment.

Wet retinoscopy yielded statistically significant differences in refractive measurements compared to Mohindra near retinoscopy in infants and children. Mohindra near retinoscopy consistently underestimated hyperopic and astigmatic refractive errors. These findings suggest that the two techniques may provide different refractive outcomes in pediatric patients. Wet retinoscopy remains an important method for pediatric refractive assessment when minimization of accommodative influences is required.

In summary, wet retinoscopy under adequate cycloplegia is recommended as the preferred method for assessing refractive error in infants and children. Ensuring complete cycloplegia is essential to improve accuracy and reduce variability in results. Mohindra near retinoscopy may be used when cycloplegia is not possible. However, its results should be interpreted with caution due to possible underestimation of hyperopia. Further research should focus on larger, multicenter studies to further compare these techniques and to evaluate the impact of partial cycloplegia on measurement differences.

##  Financial Support and Sponsorship

None.

##  Conflicts of Interest

None.
